# A chromosomal reference genome sequence for the malaria mosquito, *Anopheles gambiae*, Giles, 1902, Ifakara strain

**DOI:** 10.12688/wellcomeopenres.18854.2

**Published:** 2024-03-26

**Authors:** Tibebu Habtewold, Martin Wagah, Mgeni Mohamed Tambwe, Sarah Moore, Nikolai Windbichler, George Christophides, Harriet Johnson, Haynes Heaton, Joanna Collins, Ksenia Krasheninnikova, Sarah E. Pelan, Damon-Lee B. Pointon, Ying Sims, James W. Torrance, Alan Tracey, Marcela Uliano Da Silva, Jonathan MD Wood, Katharina von Wyschetzki, Wellcome Sanger Institute Scientific Operations: DNA Pipelines collective, Shane A. McCarthy, Daniel E. Neafsey, Alex Makunin, Mara K.N. Lawniczak

**Affiliations:** 1 Department of Life Sciences, Imperial College London, London, UK; 2 Tree of Life, Wellcome Sanger Institute, Hinxton, UK; 3 Vector Control Product Testing Unit, Ifakara Health institute, Bagamoyo, Tanzania; 4 Vector Biology Unit, Swiss Tropical and Public Health Institute, Bagamoyo, Tanzania; 5 Scientific Operations, Wellcome Sanger Institute, Hinxton, UK; 6 CSSE, Auburn University, Auburn, Alabama, USA; 7 Infectious Disease and Microbiome Program, Broad Institute, Cambridge, Massachusetts, USA; 8 Department of Immunology and Infectious Diseases, Harvard T.H. Chan School of Public Health, Boston, Massachusetts, USA

**Keywords:** Anopheles gambiae, African malaria mosquito, genome sequence, chromosomal

## Abstract

We present a genome assembly from an individual female *Anopheles gambiae* (the malaria mosquito; Arthropoda; Insecta; Diptera; Culicidae), Ifakara strain. The genome sequence is 264 megabases in span. Most of the assembly is scaffolded into three chromosomal pseudomolecules with the X sex chromosome assembled. The complete mitochondrial genome was also assembled and is 15.4 kilobases in length.

## Species taxonomy

Animalia; Arthropoda; Insecta; Diptera; Culicidae; Anophelinae; Anopheles; *Anopheles gambiae*; Giles, 1902 (NCBI txid:7165).

## Background

Twenty years ago, the African malaria mosquito *Anopheles gambiae* became the second insect to have a reference genome ^1^. This species is an important human malaria vector in Africa, and the original reference genome, which was generated from the colony known as PEST (for Pink Eye STandard) continues to be heavily used by a large community studying *Anopheles* biology. Although the PEST reference has been improved over the years, resulting in the AgamP3 assembly that remains to date ( 2; AgamP4 is AgamP3 with the mitochondrial genome included), the colony is long extinct and was a mixture of what are known today to be two incipient species: ^3^ *Anopheles gambiae sensu stricto (s.s.* or simply *An. gambiae*) and *Anopheles coluzzii*. Therefore, we sought to create an improved *An. gambiae* reference from an extant colony for the large community of users who are working on this species. Technological improvements in recent years mean we can generate reference genomes from single insects using long reads vastly improving the contiguity of the genome. Here we present a new reference genome for *An. gambiae s.s.,* sequenced as part of the *Anopheles* Reference Genomes Project (PRJEB51690). This genome derives from a single lab-reared female from an extant colony from Tanzania known as the Ifakara strain. This colony is likely to be heterokaryotypic for the 2La inversion, but the primary assembly presented here is 2L+ standard and, given the collinearity with PEST, is likely to be standard for other common inversions as well. The Ifakara strain has colonies available in Tanzania and the UK and it is available for additional labs by contacting Dr Mgeni Mohamed at Ifakara Health Institute. This new reference genome has only 33 gaps across the three chromosomes and at 264 Mb is also 39 Mb larger than the PEST chromosomal assembly (~225 Mb when excluding Ns). This is in comparison to over 6000 gaps in the PEST chromosomes, as well as a bin of contigs containing 27.3 Mb (excluding Ns) of sequences not placed on the three chromosomes. The PEST genome has been an incredibly important genomic resource for the past 20 years for the large community working on both *An. gambiae* and *An. coluzzii*, but there is now an increasing need to differentiate between these two species. The Ifakara strain reference genome will soon have an annotation available via VectorBase, and we encourage studies on *An. gambiae* to make use of this new reference genome instead of the PEST assembly.

## Genome sequence report

The genome was sequenced from a single female *An. gambiae* reared in Imperial College London, UK. The Ifakara strain was colonised by Japhet Kihonda and Bart Knols using mosquitoes collected in Njage, Tanzania (-8.234, 36.166) in 1996 ^4^. A total of 54-fold coverage in Pacific Biosciences single-molecule HiFi long reads (N50 10.760 kb) and 77-fold coverage in 10X Genomics read clouds were generated. Primary assembly contigs were scaffolded with chromosome conformation Hi-C data from a female sibling. Manual assembly curation corrected 20 missing joins (misjoins) and removed 6 retained haplotigs based on Hi-C patterns, reducing the primary assembly size by 1.0% and reducing the scaffold number by 7.8%.

**Figure 1.**
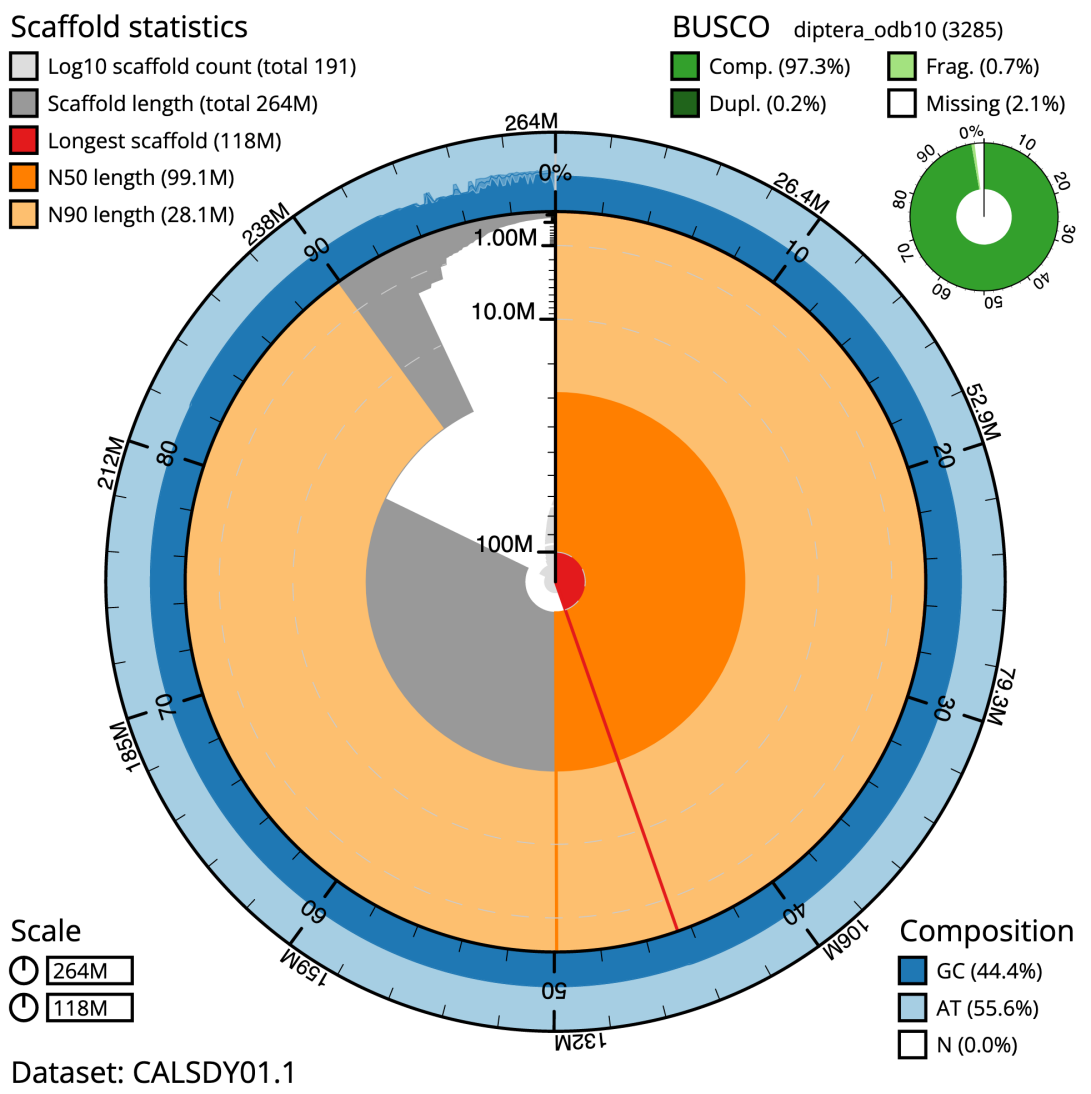
Snail plot summary of assembly statistics for *An. gambiae* assembly idAnoGamb_NW_F1_1. The main plot is divided into 1,000 size-ordered bins around the circumference with each bin representing 0.1% of the 264,466,745 bp assembly. The distribution of chromosome lengths is shown in dark grey with the plot radius scaled to the longest chromosome present in the assembly (118,196,952 bp, shown in red). Orange and pale-orange arcs show the N50 and N90 chromosome lengths (99,149,756 and 28,097,889 bp), respectively. The pale grey spiral shows the cumulative chromosome count on a log scale with white scale lines showing successive orders of magnitude. The blue and pale-blue area around the outside of the plot shows the distribution of GC, AT and N percentages in the same bins as the inner plot. A summary of complete, fragmented, duplicated and missing BUSCO genes in the diptera_odb10 set is shown in the top right. An interactive version of this figure is available at https://blobtoolkit.genomehubs.org/view/idAnoGambNW_F1_1/dataset/CALSDY01.1/snail.

**Figure 2.**
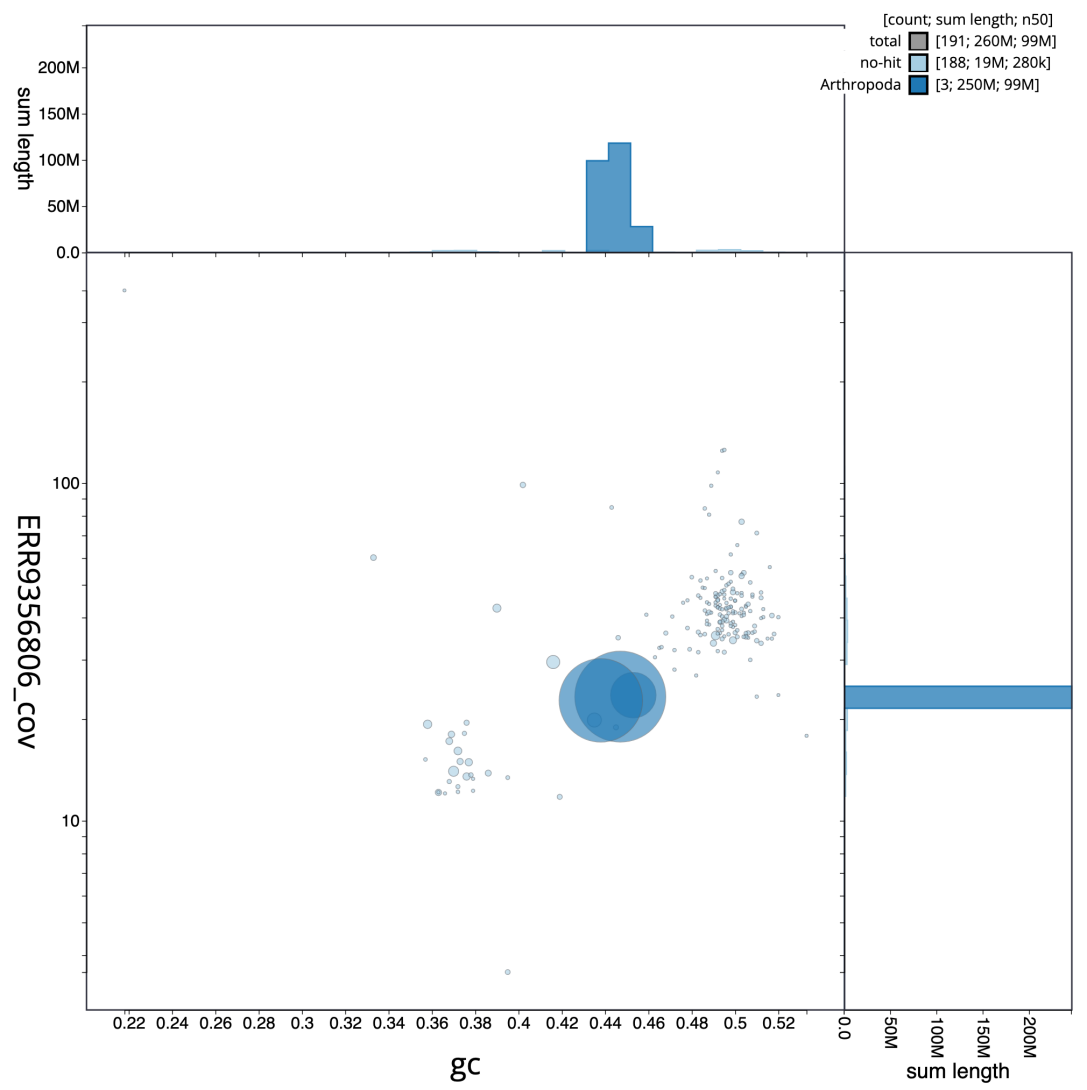
Blob plot of base coverage in a subset of idAnoGambNW_F1_1 10x linked reads against GC proportion for *An. gambiae* assembly idAnoGambNW_F1_1. Chromosomes are coloured by phylum. Circles are sized in proportion to chromosome length. Histograms show the distribution of chromosome length sum along each axis. An interactive version of this figure is available at https://blobtoolkit.genomehubs.org/view/idAnoGambNW_F1_1/dataset/CALSDY01.1/blob.

**Figure 3.**
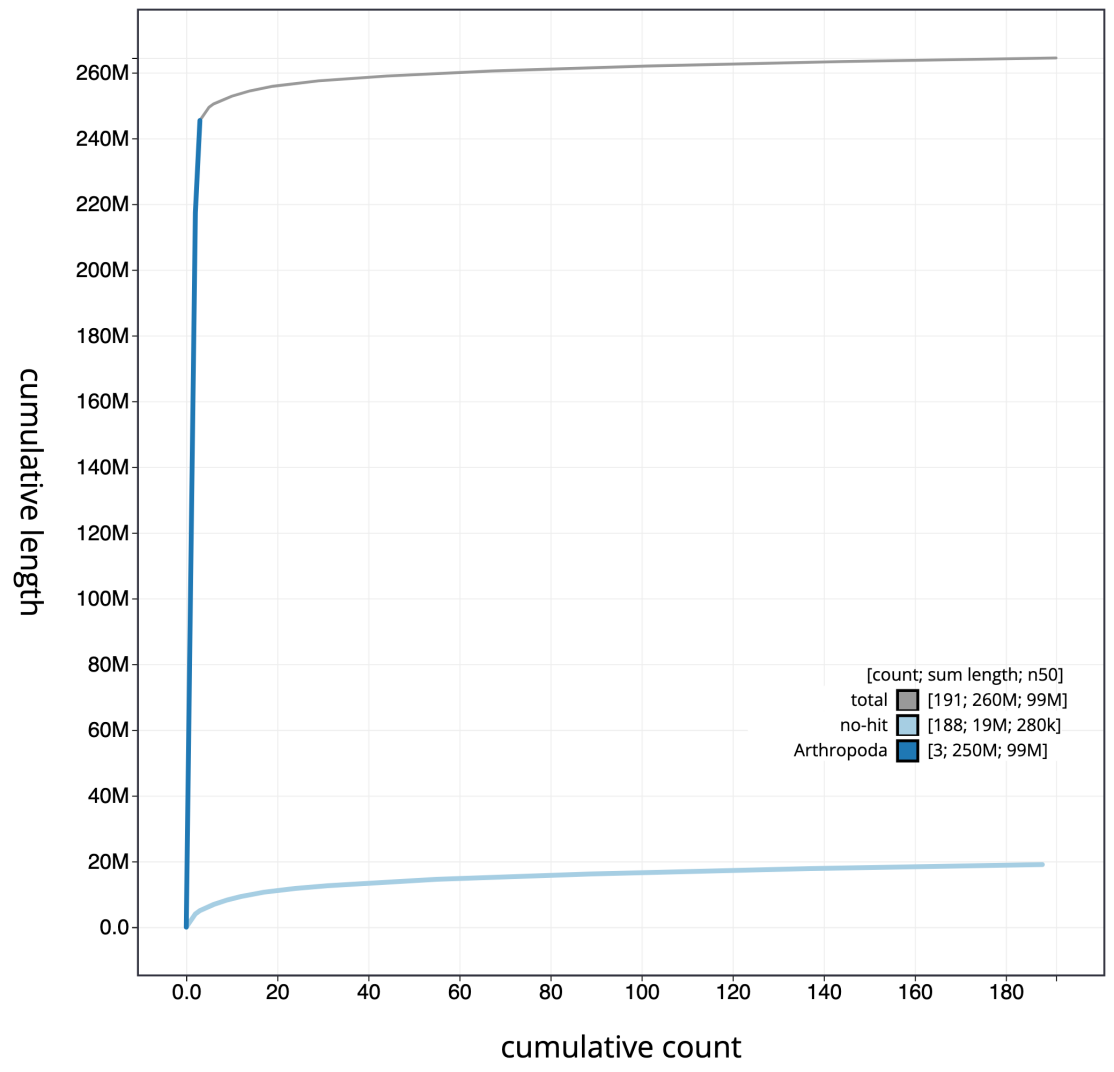
Cumulative chromosome length for *An. gambiae* assembly idAnoGambNW_F1_1. The grey line shows cumulative length for all chromosomes. Coloured lines show cumulative lengths of chromosomes assigned to each phylum using the buscogenes taxrule. The interactive version of this figure is available at https://blobtoolkit.genomehubs.org/view/idAnoGambNW_F1_1/dataset/CALSDY01.1/cumulative.

**Figure 4.**
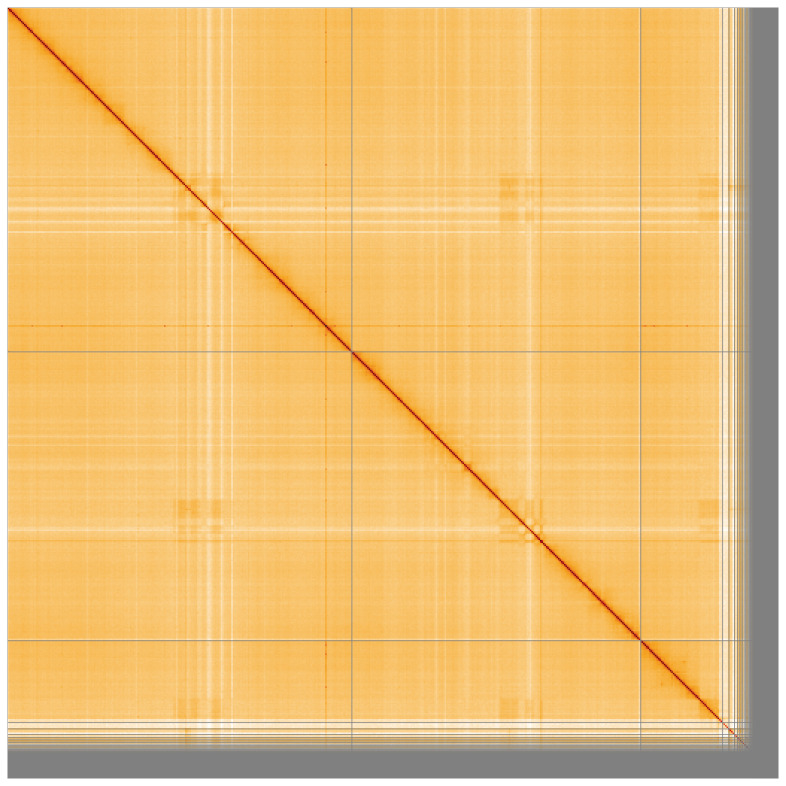
Genome assembly of *An. gambiae*, idAnoGambNW_F1_1: Hi-C contact map. Visualised in HiGlass. Chromosomes order: 2RL, 3RL, X, then remaining scaffolds. The interactive Hi-C map can be viewed at https://genome-note-higlass.tol.sanger.ac.uk/l/?d=Qbx2q2EPRIuiC3qXX8BfFA.

**Figure 5.**
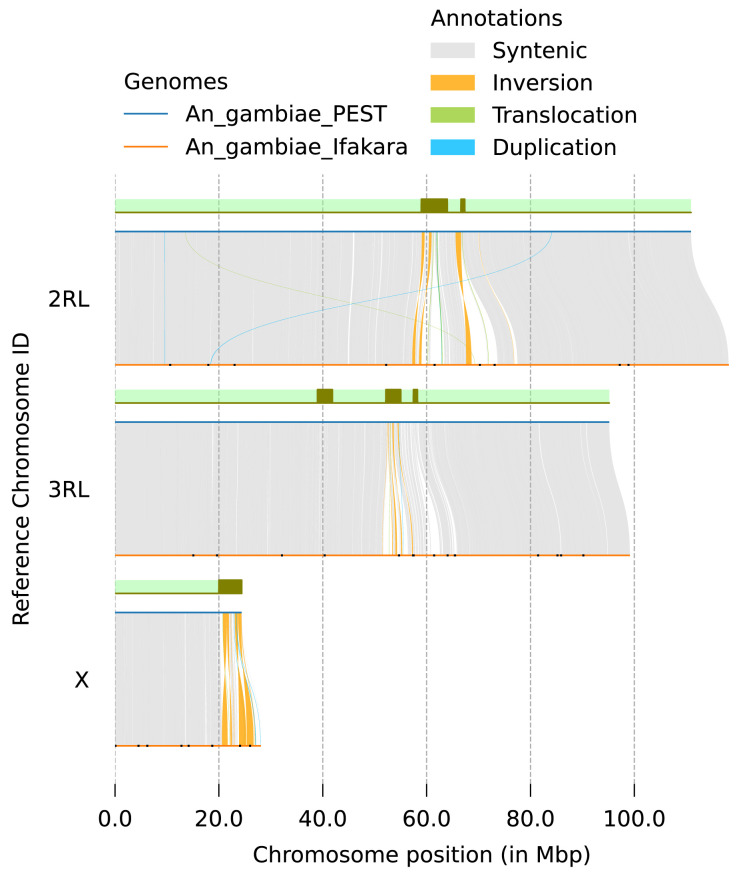
Synteny between genome assemblies of *An. gambiae*, AgamP3 (PEST) and idAnoGambNW_F1_1 (Ifakara). Grey rectangles on green background represent positions of pericentric and intercalary heterochromatin in AgamP3 ^8^. Remaining gaps in idAnoGambNW_F1_1 are indicated with black dots.

The final assembly has a total length of 264 Mb in 191 sequence scaffolds with a scaffold N50 of 99.150 Mb ( Table 1, Figure 1). 92.29% of the assembly sequence was assigned to three chromosomal-level scaffolds, representing two autosomes and the X chromosome named and oriented against the AgamP3 assembly ^2^ (GCF_000005575.2) ( Figure 2, Figure 3; Table 2). Hi-C contact map demonstrates overall agreement with the assembly with some contact dropouts in regions that are either repetitive or diverged between samples used for PacBio and Hi-C ( Figure 4). Synteny analysis against the AgamP3 assembly revealed overall collinearity between the genomes and significant increase in sequence recovery in heterochromatic regions ( Figure 5). The total number of assembly gaps across the three chromosomes was reduced dramatically from 6,302 in PEST (AgamP3) to 33 in our assembly ( Figure 5, Table 2).

**Table 1.** Genome data for *An. gambiae*, idAnoGambNW_F1_1.

*Project accession data*	
Assembly identifier	idAnoGambNW_F1_1
Species	*Anopheles gambiae*
Specimen	idAnoGambNW-F1_1
NCBI taxonomy ID	7165
BioProject	PRJEB53260
BioSample ID	ERS10527367
Isolate information	female, whole organism
*Raw data accessions*	
PacificBiosciences SEQUEL II	ERR9439502
10X Genomics Illumina	ERR9356803, ERR9356804, ERR9356805, ERR9356806
Hi-C Illumina	ERR9356802
PolyA RNA-Seq Illumina	ERR9356809, ERR9356810
*Genome assembly*	
Assembly accession	GCA_943734735
*Accession of alternate haplotype*	GCA_943734675
Span (Mb)	264.467
Number of contigs	232
Contig N50 length (Mb)	10.625
Number of scaffolds	191
Scaffold N50 length (Mb)	99.150
Longest scaffold (Mb)	118.197
BUSCO * genome score	C:97.3%[S:97.1%,D:0.2%], F:0.7%,M:2.1%,n:3285

* BUSCO scores based on the diptera_odb10 BUSCO set using BUSCO 5.3.2. C=complete [S=single copy, D=duplicated], F=fragmented, M=missing, n=number of orthologues in comparison. A full set of BUSCO scores is available at https://blobtoolkit.genomehubs.org/view/idAnoGambNW_F1_1/dataset/CALSDY01.1/busco.

**Table 2.** Chromosomal pseudomolecules in the genome assembly of *An. gambiae*, idAnoGambNW_F1_1.

INSDC accession	Chromosome	Size (Mb)	Count	Gaps
OX030907.1	2RL	118.197	1	9
OX030908.1	3RL	99.150	1	15
OX030909.1	X	28.098	1	9
OX030910.1	MT	0.015	1	0
	X Unlocalised	11.519	161	2
	Unplaced	7.487	26	6

**Figure 6.**
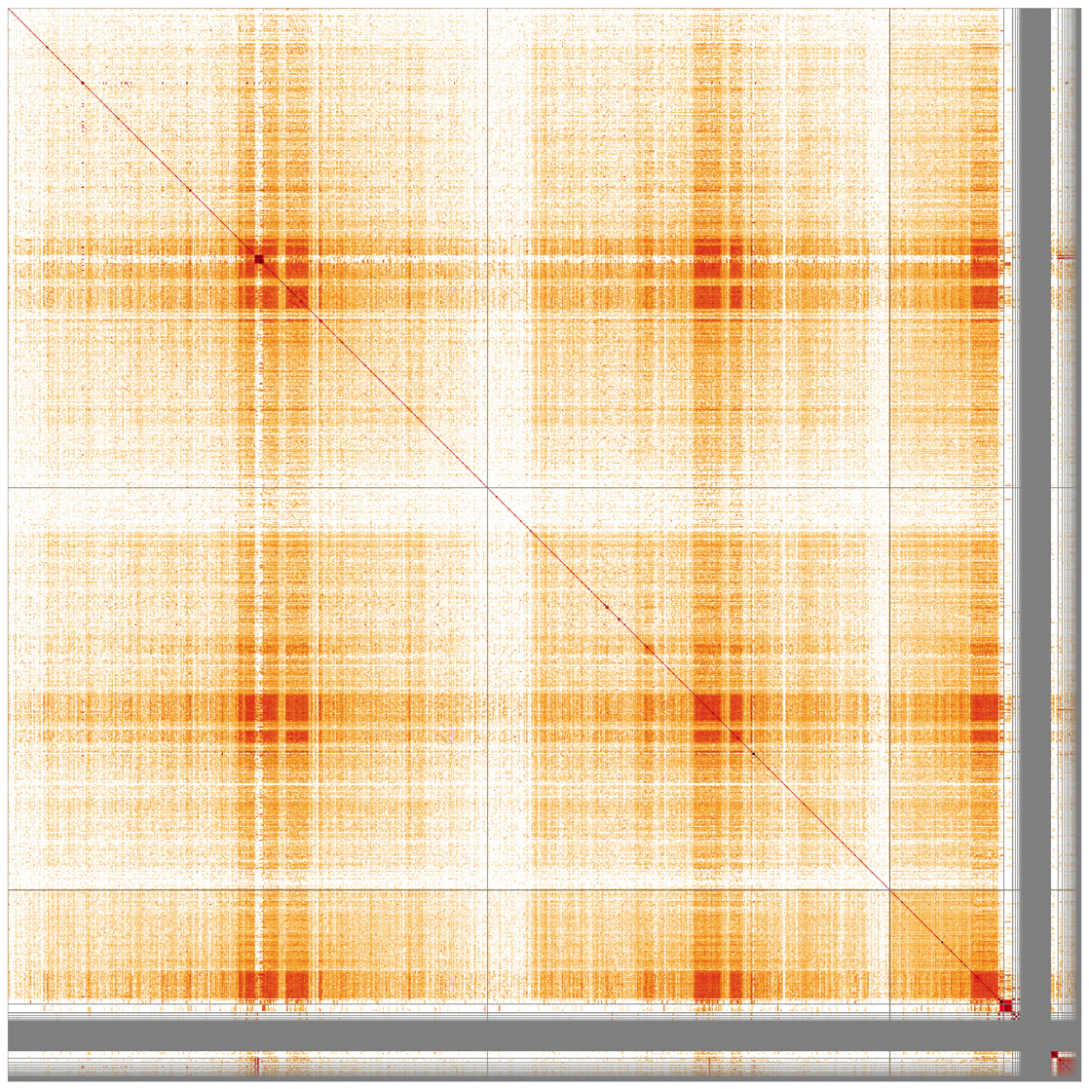
Genome assembly of *An. gambiae*, idAnoGambNW_F1_1 sequence similarity heatmap. Produced with StainedGlass, visualised in HiGlass. Chromosomes order: 2RL, 3RL, X, followed by the unassembled contigs. Darker colours represent higher sequence similarity, notably at pericentric heterochromatin.

Chromosome arms, candidate centromere sequences, and the rDNA region were delineated based on the presence of characteristic tandem repeat arrays ( Figure 6; Table 3). The candidate centromere of 2RL comprised interspersed blocks of pericentriс repeat Ag93 ^5^ and variable transposon-derived tandem repeats. The candidate centromere of 3RL comprised only a single block of Ag93 and was much shorter than the one in 2RL, most likely indicating an assembly collapse in this region. The candidate centromere of X comprised a single block of diverged variants Ag113, the only following fragment being the rDNA block starting at X:28,005,454. Other putative centromere associated repeats, e.g. autosomal AgY53C and X-linked AgX367 ^6^, were only found within unassembled contigs, indicating that some of the most highly repetitive genomic regions remain to be assembled to chromosomal state.

**Table 3.** Chromosomal arms in the genome assembly of *An. gambiae*, idAnoGambNW_F1_1.

Arm	Chromosome	Start	End
2R	2RL	1	60,921,823
2L	2RL	62,889,739	118,196,952
3R	3RL	1	54,661,593
2L	3RL	54,724,984	99,149,756
X	X	1	27,206,008

Gene annotation was performed by RefSeq team in NCBI (accession GCF_943734735.2). A total of 15,165 genes were predicted, including 12,519 protein-coding genes and 3,148 non-coding RNAs. 

The assembly has a BUSCO 5.3.2 ^7^ completeness of 97.3% using the diptera_odb10 reference set. While not fully phased, the assembly deposited is of one haplotype. Contigs corresponding to the second haplotype have also been deposited.

## Methods

### Sample acquisition and nucleic acid extraction

*Anopheles gambiae* offspring were reared from a lab-reared gravid female of Ifakara strain by Tibebu Habtewold. A single female idAnoGambNW-F1_1 was used for Pacific BioSciences and 10x genomics, and its sibling female idAnoGambNW-F1_3 was used for Arima Hi-C, as described below.

For high molecular weight (HMW) DNA extraction, one whole insect (idAnoGambNW-F1_1) was disrupted by manual grinding with a blue plastic pestle in Qiagen MagAttract lysis buffer and DNA was extracted using the Qiagen MagAttract HMW DNA extraction kit with two minor modifications including halving the volumes recommended by the manufacturer due to small sample size ( *Anopheles* mosquitoes typically weigh 2–3 mg) and running two elution steps of 100 μl each to increase DNA yield. The quality of the DNA was evaluated using an Agilent FemtoPulse to ensure that most DNA molecules were larger than 30 kb, and preferably >100 kb. In general, single *Anopheles* extractions range in total estimated DNA yield from 200 ng to 800 ng, with an average yield of 500 ng. Low molecular weight DNA was removed using an 0.8X AMpure XP purification. A small aliquot (less than ~5% of the total volume) of HMW DNA was set aside for 10X Linked Read sequencing and the rest of the DNA was sheared to an average fragment size of 12–20 Kb using a Diagenode Megaruptor 3 at speeds ranging from 27 to 30. Sheared DNA was purified using AMPure PB beads with a 1.8X ratio of beads to sample to remove the shorter fragments and concentrate the DNA sample. The concentration and quality of the sheared and purified DNA was assessed using a Nanodrop spectrophotometer and Qubit Fluorometer with the Qubit dsDNA High Sensitivity Assay kit. Fragment size distribution was evaluated by running the sheared and cleaned sample on the FemtoPulse system once more. The median DNA fragment size for *Anopheles* mosquitoes was 15 kb and the median yield of sheared DNA was 200 ng, with samples typically losing about 50% of the original estimated DNA quantity through the process of shearing and purification.

For Hi-C data generation, a separate sibling mosquito specimen (idAnoGambNW-F1_3) was used as input material for the Arima V2 Kit according to the manufacturer’s instructions for animal tissue. This approach of using a sibling was taken in order to enable all material from a single specimen to contribute to the PacBio data generation given we were not always able to meet the minimum suggested guidance of starting with > 300 ng of HMW DNA from a specimen. Samples proceeded to the Illumina library prep stage even if they were suboptimal (too little tissue) going into the Arima reaction.

To assist with annotation, which will be made available through VEuPathDB VectorBase in due course, RNA was extracted from separate whole sibling insect specimens (idAnoGambNW-F1_9 and idAnoGambNW-F1_10) using TRIzol, according to the manufacturer instructions. RNA was then eluted in 50 μl RNAse-free water, and its concentration was assessed using a Nanodrop spectrophotometer and Qubit Fluorometer using the Qubit RNA Broad-Range (BR) Assay kit. Analysis of the integrity of the RNA was done using Agilent RNA 6000 Pico Kit and Eukaryotic Total RNA assay. Samples were not always ideally preserved for RNA, so qualities varied but all were sequenced anyway.

### Sequencing

We prepared libraries as per the PacBio procedure and checklist for SMRTbell Libraries using Express TPK 2.0 with low DNA input. Every library was barcoded to support multiplexing. Final library yields ranged from 20 ng to 100 ng, representing only about 25% of the input sheared DNA. Libraries from two specimens were typically multiplexed on a single 8M SMRT Cell. Sequencing complexes were made using Sequencing Primer v4 and DNA Polymerase v2.0. Sequencing was carried out on the Sequel II system with a 24-hour run time and a 2-hour pre-extension. A 10X Genomics Chromium read cloud sequencing library was also constructed according to the manufacturer’s instructions (this product is no longer available). Only 0.5 ng of DNA was used and only 25–50% of the gel emulsion was put forward for library prep due to the small genome size. For Hi-C data generation, following the Arima HiC V2 reaction, samples were processed through Library Preparation using a NEB Next Ultra II DNA Library Prep Kit and sequenced aiming for 100x depth. RNA libraries were created using the directional NEB Ultra II stranded kit. Sequencing was performed by the Scientific Operations core at the Wellcome Sanger Institute on Pacific Biosciences SEQUEL II (HiFi), Illumina NovaSeq 6000 (10X and Hi-C), or Illumina HiSeq 4000 (RNAseq).

### Genome assembly

Assembly was carried out with Hifiasm ^9^; haplotypic duplications were identified and removed with purge_dups ^10^. One round of polishing was performed by aligning 10X Genomics read data to the assembly with longranger align, calling variants with freebayes ^11^. The assembly was then scaffolded with Hi-C data ^12^ using SALSA2 ^13^. The assembly was checked for contamination as described previously ^14^. Manual curation was performed using gEVAL ^15^, HiGlass ^16^ and Pretext ^17^. The mitochondrial genome was assembled using MitoHiFi ^18^, which performs annotation using MitoFinder ^19^. The genome was analysed and BUSCO scores were generated within the BlobToolKit environment ^20^. Synteny analysis was performed with syri ^21^ and visualised with plotsr ^22^. Repetitive sequences were visualised with StainedGlass ^23^ and tandem repeats were annotated with ULTRA ^24^. Table 4 contains a list of all software tool versions used, where appropriate.

**Table 4.** Software tools used.

Software tool	Version	Source
hifiasm	0.14	9
purge_dups	1.2.3	10
SALSA2	2.2-4c80ac1	13
longranger align	2.2.2	25
freebayes	1.3.1	11
MitoHiFi	2	18
gEVAL	N/A	15
HiGlass	1.11.6	16
PretextView	0.1.x	17
BlobToolKit	3.4.0	20
BUSCO	5.3.2	7
syri	1.6	21
plotsr	0.5.3	22
StainedGlass	0.5	23
ULTRA	1.0.0 beta	24

### Ethics/compliance issues

The genetic resources accessed and utilised under this project were done so in accordance with the UK ABS legislation (Nagoya Protocol (Compliance) (Amendment) (EU Exit) Regulations 2018 (SI 2018/1393)) and the national ABS legislation within the country of origin, where applicable.

## Data Availability

NCBI BioProject: *Anopheles gambiae* genome assembly, idAnoGambNW_F1_1. Accession number PRJEB53260; https://identifiers.org/bioproject/PRJEB53260 ^26^. The genome sequence is released openly for reuse. The *Anopheles gambiae* genome sequencing initiative is part of the Anopheles Reference Genomes project PRJEB51690. All raw sequence data and the assembly have been deposited in INSDC databases. Raw data and assembly accession identifiers are reported in Table 1.

## Author information

Members of Wellcome Sanger Institute Scientific Operations: DNA Pipelines collective are listed here: https://doi.org/10.5281/zenodo.4790455.
