# Supplementary material for: A chromosomal reference genome sequence for the malaria mosquito, Anopheles gambiae, Giles, 1902, Ifakara strain
Source: Wellcome Open Res. 2024 Mar 26;8:74. Originally published 2023 Feb 13. [Version 2] doi: 10.12688/wellcomeopenres.18854.2 (PMC10326452; doi:10.12688/wellcomeopenres.18854.2)
Supplement: Peer review history

# Reviewer report: Reviewer response for version 1

**DOI:** 10.21956/wellcomeopenres.20906.r61374

**Published:** 2023-07-06

**Authors:** Jason Miller

**Affiliations:** 1 Computers, Mathematics, Engineering, Shepherd University, Shepherdstown, West Virginia, USA

This data note describes an assembly of the malaria mosquito *Anopheles gambiae*. The new assembly improves upon, and will likely replace, the existing reference. Unlike the prior reference, the new sequence is based on DNA from a single individual. Despite the small quantity of available DNA, the authors heroically applied hifi long read, 10X composite read, and HiC technology. The paper does not include biological inference or even gene annotation beyond BUSCO analysis, but this is in accordance with the project goals to release assemblies quickly, and the journal’s instructions for data notes.

 Minor Revisions Suggested:

 The paper should clarify which assembly statistics were generated after the manual curation exclusively. For example, if the manual removal of 6 haplotigs was informed by pre-curation BUSCO duplicated gene analysis, then the post-curation BUSCO results should be understood to be a curation objective that was met, and not an independent validation.

 The background should clear up the reference nomenclature and history. In this version, the background refers to PEST exclusively, but the next section refers to AgamP3, and later to “PEST (AgamP3)”. The background should not say PEST was “incredibly important” (twice). It would be more convincing to cite discoveries enabled by PEST. The background should clarify the sources of its claims that “the colony has since become extinct” and that “it became clear that it was a mixture” of species. The authors should cite or explain “the 2La inversion” and why they believe “the colony is likely to be heterokaryotypic.”

 The paper could briefly compare its methods and results to those of the Anopheles 16 genomes project (Neafsey 2013) ^1^, the Anopheles 1000 genomes project (Genome Research 2020) ^2^, and other chromosome-scale mosquito assemblies (e.g. Ghurye et al 2019 ^3^ and Ayala et al 2022 ^4^). INSDC could be referenced (Brunak 2002 ^5^).

 Figure 5 and Table 2 are clear, helpful, and impressive. Figures 1 to 4 are unhelpful just because the whole genome is in 4 scaffolds. For example, the complicated snail plot is just a polar chart of the four scaffold lengths from Table 2. Figure 4 shows nearly uniform background noise, which looks good, but it is hard to interpret quantitatively without a color legend or a comparative example. It seems this paper follows the manuscript template used by other products of this project. I would not remove the figures, but I would add text to tell the reader what the figures show. In this version, the text links to the figures without explanation and the captions give only technical details.

Are sufficient details of methods and materials provided to allow replication by others?

Yes

Is the rationale for creating the dataset(s) clearly described?

Yes

Are the datasets clearly presented in a useable and accessible format?

Yes

Are the protocols appropriate and is the work technically sound?

Yes

Reviewer Expertise:

Bioinformatics, genomics, transcriptomics, machine learning.

I confirm that I have read this submission and believe that I have an appropriate level of expertise to confirm that it is of an acceptable scientific standard.

## References

[1] : The evolution of the Anopheles 16 genomes project. *G3 (Bethesda)* .2013;3(7) : 10.1534/g3.113.006247 1191-4 10.1534/g3.113.006247 23708298 PMC3704246

[2] : Genome variation and population structure among 1142 mosquitoes of the African malaria vector species Anopheles gambiae and Anopheles coluzzii. *Genome Res* .2020;30(10) : 10.1101/gr.262790.120 1533-1546 10.1101/gr.262790.120 32989001 PMC7605271

[3] : A chromosome-scale assembly of the major African malaria vector Anopheles funestus. *Gigascience* .2019;8(6) : 10.1093/gigascience/giz063 10.1093/gigascience/giz063 31157884 PMC6545970

[4] : The genome sequence of the malaria mosquito, Anopheles funestus, Giles, 1900. *Wellcome Open Research* .2022;7: 10.12688/wellcomeopenres.18445.1 10.12688/wellcomeopenres.18445.1 36874567 PMC9975407

[5] : Nucleotide Sequence Database Policies. *Science* .2002;298(5597) : 10.1126/science.298.5597.1333b 10.1126/science.298.5597.1333b 12436968

# Reviewer report: Reviewer response for version 1

**DOI:** 10.21956/wellcomeopenres.20906.r58446

**Published:** 2023-06-12

**Authors:** Alexander Franz, Zachary Speth

**Affiliations:** 1 University of Missouri, Columbia, Missouri, USA

Presented is the highest quality genome assembly for *An. gambiae s.s.* generated to date. With the application of three long read sequencing methods, Habtewold *et al.* constructed chromosome length assemblies with coverage of the two *An. gambiae* autosomes 2RL and 3RL, as well as the X chromosome and the mitochondrial chromosome. This improved reference genome is a highly useful resource to any researcher currently studying *An. gambiae*.

 The most advanced/appropriate sequencing methods were used in this work. The overall coverage level of >50-fold (single-molecule HIFi long reads) and >70-fold in 10xGenomics reads looks sufficient to reliably distinguish SNPs between haplotypes. The genome data are available at NCBI BioProject. The data are accessible as checked for the X chromosome (as an example). Figures 2, 3, and 4 are not easy to understand for a non-specialist.

Are sufficient details of methods and materials provided to allow replication by others?

Yes

Is the rationale for creating the dataset(s) clearly described?

Yes

Are the datasets clearly presented in a useable and accessible format?

Yes

Are the protocols appropriate and is the work technically sound?

Yes

Reviewer Expertise:

mosquito transgenesis, transcriptome/proteome analysis, single-cell transcriptomics

We confirm that we have read this submission and believe that we have an appropriate level of expertise to confirm that it is of an acceptable scientific standard.
